# AAV-mediated base editing restores cochlear gap junction in GJB2 dominant-negative mutation-associated syndromic hearing loss model

**DOI:** 10.1172/jci.insight.185193

**Published:** 2025-03-10

**Authors:** Takao Ukaji, Daisuke Arai, Harumi Tsutsumi, Ryoya Nakagawa, Fumihiko Matsumoto, Katsuhisa Ikeda, Osamu Nureki, Kazusaku Kamiya

**Affiliations:** 1 Department of Otorhinolaryngology, Juntendo University Faculty of Medicine, Tokyo, Japan.; 2 Department of Biological Sciences, Graduate School of Science, The University of Tokyo, Tokyo, Japan.

**Keywords:** Genetics, Therapeutics, Gene therapy

## Abstract

Mutations in the gap junction β2 (*GJB2*) gene, which encodes connexin 26, are the leading cause of genetic deafness. These mutations are characterized by the degeneration and fragmentation of gap junctions and gap junction plaques (GJPs) composed of connexin 26. Dominant-negative mutations of *GJB2*, such as R75W, cause syndromic hearing loss and palmoplantar keratoderma. We previously reported that the R75W mutation, a single-base substitution where C is replaced by T, causes fragmentation of GJPs. Therefore, an adenine base editor (ABE), which enables A-to-G base conversions, can potentially be useful for the treatment of this genetic disease. Here, we report that an all-in-one adeno-associated virus (AAV) vector, which includes a compact ABE (SaCas9-NNG-ABE8e) with broad targeting range, and a sgRNA targeting the R75W mutation in *GJB2* corrected this pathogenic mutation and facilitated the recovery of the gap junction intercellular communication network of GJPs. In a transgenic mouse model with the *GJB2* R75W mutation, AAV-mediated base editing also restored the fragmented GJPs to orderly outlines in cochlear supporting cells. Our findings suggest that an ABE-based base-editing strategy could be an optimal treatment for the dominant form of *GJB2*-related hearing loss, *GJB2*-related skin diseases, and other deafness-related mutations, especially single-base substitutions.

## Abstract 2

AAV-mediated base editing therapy enables to repair effectively the pathogenic gap junction plaques (GJPs) and restore its physiological function in dominant-negative *GJB2*-related hearing loss.

## Graphical abstract

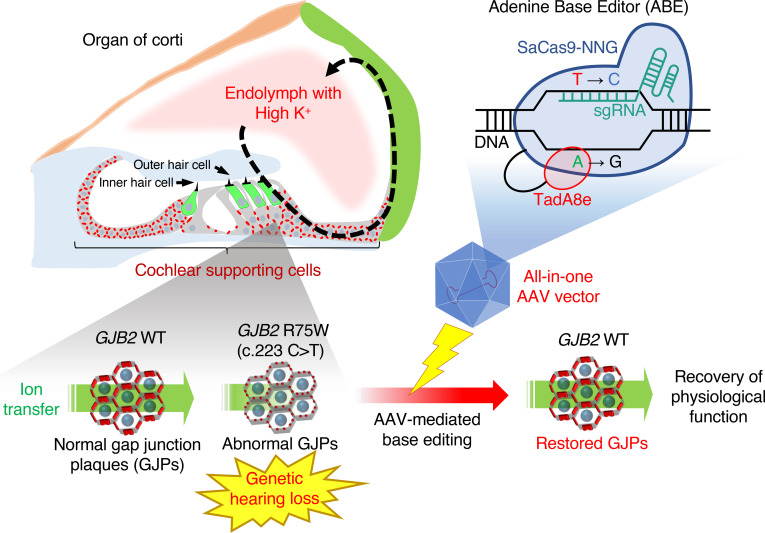

## Introduction

Hearing loss is the most common congenital sensory deficit (1), affecting approximately 1 in 1,000 children at birth or during early childhood. Approximately half of these cases of severe hearing loss (termed prelingual deafness) (2, 3) are attributable to genetic causes (4). To date, nearly 200 deafness-related loci and causative genes within these loci have been identified (https://hereditaryhearingloss.org/); by far the most common and best-characterized gene is gap junction β2 (*GJB2*; OMIM 121011), which encodes connexin 26 (CX26) (5). CX26 is a critical component of cochlear nonsensory cells, including cochlear supporting cells, as well as cochlear structures, such as the spiral limbus, stria vascularis, and spiral ligament, where it contributes to the formation of intercellular gap junctions (6–11). Tens to thousands of gap junctions cluster into semicrystalline arrays to form gap junction plaques (GJPs) (12). GJPs assemble to form channels between cochlear supporting cells, allowing the rapid removal of K^+^ from the base of hair cells and the recycling of K^+^ back to the endolymph to maintain cochlear homeostasis (13).

We previously demonstrated that a mutation in *GJB2* resulted in a drastic disruption of the CX26/CX30 macromolecular complex and decreased gap junction intercellular communication (GJIC) in the cochlea (14). We also reported that adeno-associated virus–mediated (AAV-mediated) delivery of *GJB2* to cochlea significantly improved the auditory responses in a *Gjb2* conditional knockout mouse model both perinatally (15) and in adults (our unpublished observations). Normal hearing requires CX26, and thus hearing loss caused by recessive inheritance of a mutant *GJB2* might be cured by functional replacement with WT *GJB2*. In contrast, for hearing loss caused by a dominant-negative mutation in *GJB2*, genome editing to restore normal gene function rather than *GJB2* replacement is considered to be the best approach to treatment.

The dominant-negative mutation R75W in *GJB2*, which arises de novo during early embryogenesis, underlies syndromic hearing loss as well as deafness with palmoplantar keratoderma and is detected in 0.09% of probands with sensorineural hearing loss in Han Chinese populations (16). Xenopus oocyte expression system demonstrated that R75W mutants not only failed to show channel-forming activity, but also blocked the activity of coexpressed WT CX26 (CX26 WT), implying a dominant-negative effect (17). Our previous report also demonstrated that transgenic (TG) mice carrying the dominant-negative mutation R75W in *GJB2* (CX26*^R75W+^*) showed drastically fragmented and small vesicle–like GJPs in cochlear cells (14).

CRISPR-mediated base editors, comprising the Cas9 nickase fused to an adenine or cytosine deaminase, enable A-to-G or C-to-T substitutions at target genomic sites, and thus have been harnessed for the treatment of point mutations, which represent the largest class of pathogenic mutations (18). Notably, ABE8e, which consists of the *Streptococcus pyogenes* Cas9 (SpCas9) D10A nickase fused to the engineered tRNA-specific adenosine deaminase (TadA8e), exhibits high A-to-G base conversion efficiencies and low off-target efficiencies (19). A recent study demonstrated that dual AAV-mediated delivery of an ABE8e to the retina corrected a pathogenic mutation in a retinal degradation, protected against photoreceptor degeneration, and rescued visual function in the rd10 mouse model of retinitis pigmentosa (20). We recently developed a capsid-modified AAV vector (AAV-Sia6e) with high infection tropism for Deiters’ cells, Hensen’s cells, inner and outer sulcus cells, and fibrocytes expressing the *GJB2* gene, and its AAV-mediated efficient delivery of the *GJB2* gene improved the auditory responses in an adult mouse model of *Gjb2*-related deafness (our unpublished observations). Since the R75W mutation in *GJB2* is a single-base substitution from C to T, our AAV vector equipped with ABE8e has the potential to mediate an A-to-G base conversion at the R75W mutation, thereby restoring the abnormal formation of GJPs. However, conventional ABE8e cannot be packaged into a single AAV vector due to its large gene size, hampering clinical applications to in vivo gene therapy (21).

In the present study, we combined a compact adenine base editor (ABE) with an AAV-Sia6e vector to develop a potentially novel all-in-one AAV vector platform for restoring functionality to abnormal GJPs caused by *GJB2* mutations. This platform successfully induces the T-to-C (A-to-G) base conversion at the R75W mutation in mammalian cells. Furthermore, it repaired fragmented GJPs and recovered their physiological function in mouse models with the *GJB2* R75W mutation, which causes severe hearing loss. Collectively, our findings provide a therapeutic approach for a *GJB2* dominant-negative mutation that is not possible with gene-replacement therapy.

## Results

### In vitro screening of ABEs targeting the mutation R75W in GJB2.

**Figure 1:**
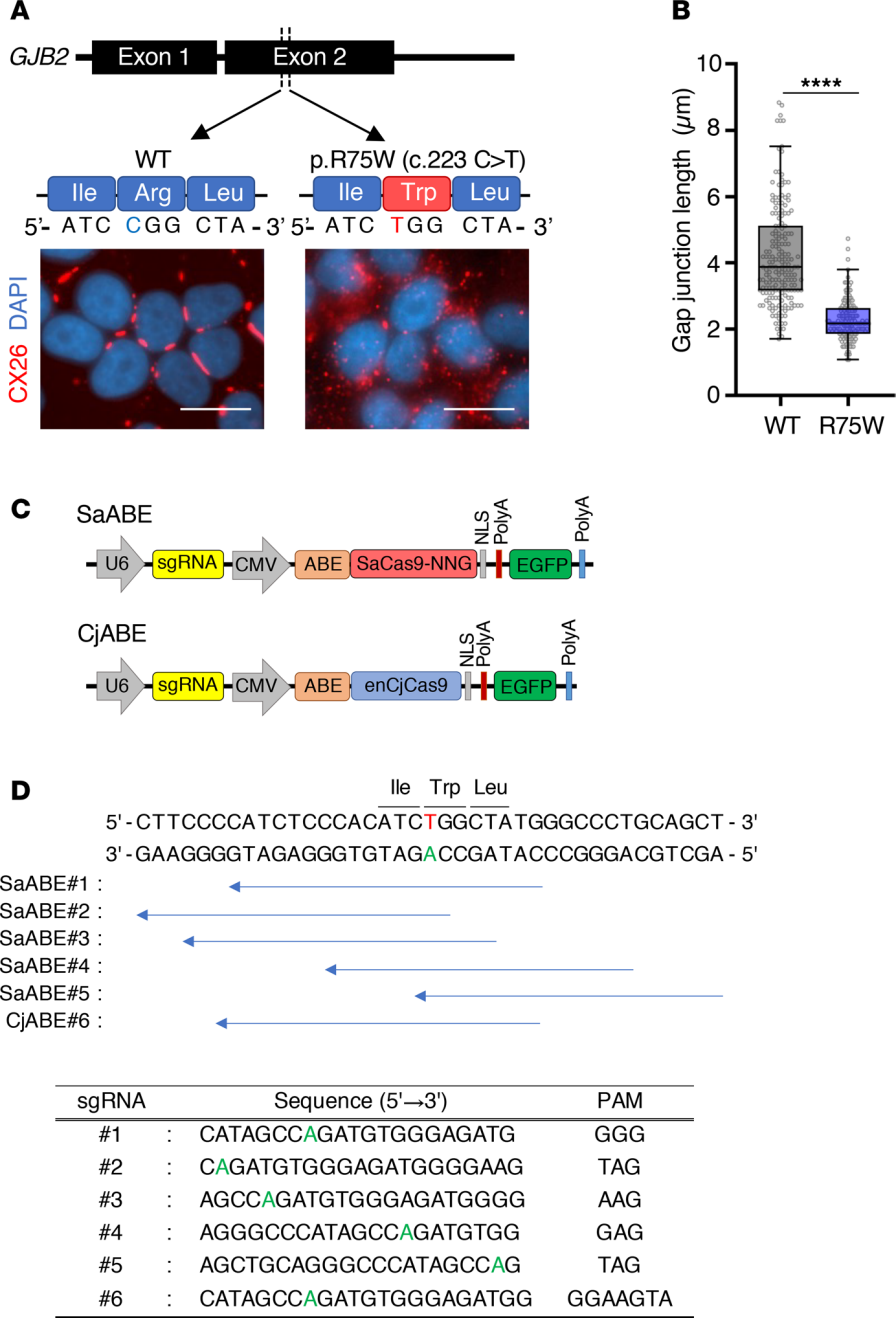
Generation of a base-editing vector for GJB2 R75W. (**A**) Representative images of GJPs in which CX26 is encoded by the normal GJB2 gene (left) or the CX26 mutant with a missense mutation in the *GJB2* gene (c.223C to T, pR75W) (right). Scale bar: 10 μm. (**B**) Length of GJPs from CX26 WT and R75W. The box-and-whisker plot shows median, interquartile range, and minimum and maximum values; isolated dots beyond the whiskers correspond to outliers defined as a value that is smaller than the lower quartile −1.5 × the interquartile range or larger than the upper quartile +1.5 times the interquartile range. *n* = 190 for WT and R75W. Statistical significance was determined with the Mann-Whitney *U* test. ****P < 0.0001. (**C**) Schematic representation of the SaABE and CjABE constructs. (**D**) Six sgRNAs were designed to substitute the target mutation (red letter) within the ABE8e activity window (blue arrows).

**Figure 2:**
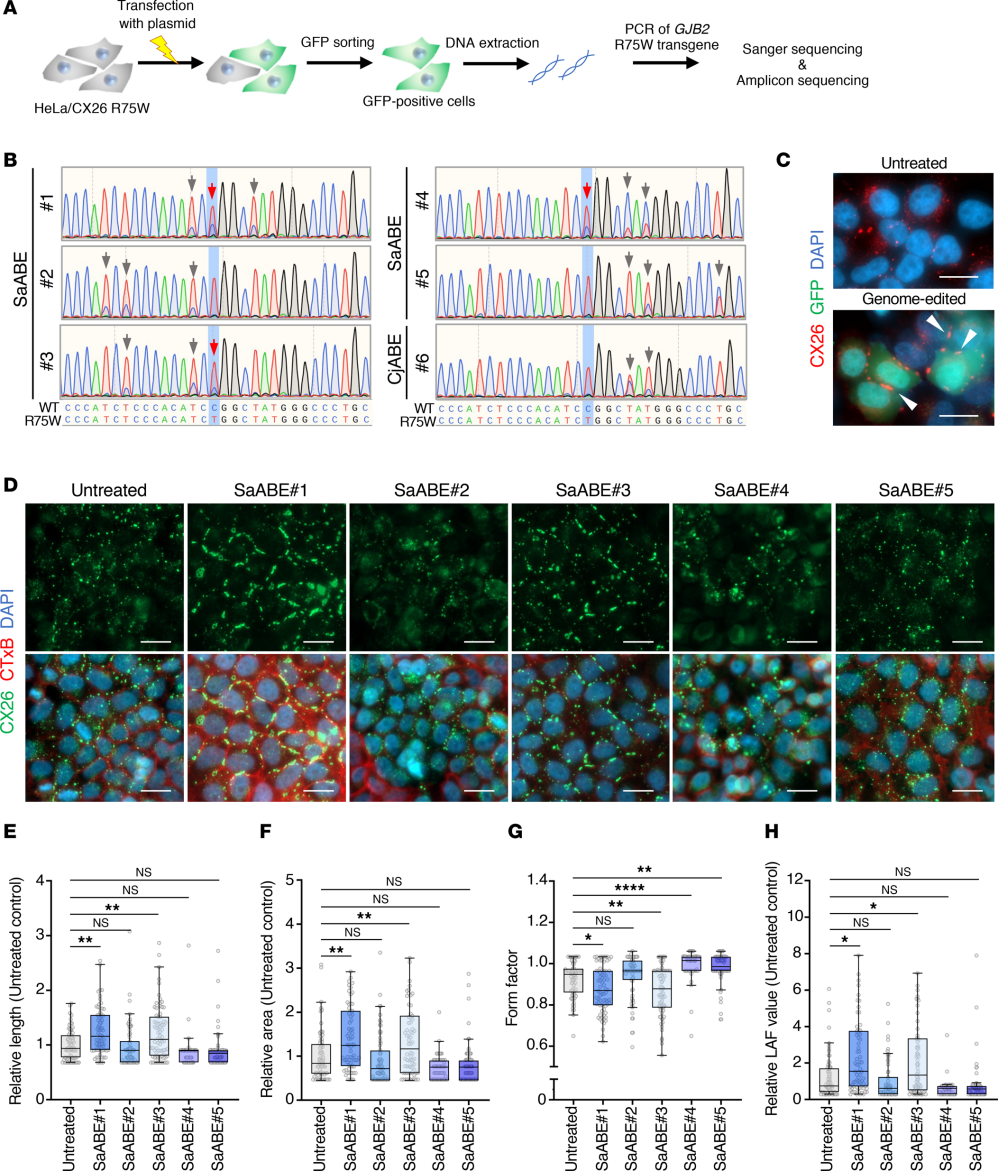
Analysis of the base-editing efficacy. (**A**) Strategy for assessing the base-editing efficiency for the mutation *GJB2* R75W. (**B**) Representative results from direct genome sequencing after base editing via SaABE#1 to #5 or CjABE#6. The sequence at the bottom is the forward sequence. Red arrows indicate correction of the target site, and gray arrows indicate bystander editing. *n* = 3 technical replicates, representative of 3 independent experiments. (**C**) Representative images of EGFP^+^ cells expressing CX26 (red) and forming GJPs after base-editing plasmid transfection. Arrowheads indicate extensive GJPs in EGFP-expressing cells after base editing. Scale bar: 10 μm. (**D**) Representative images of GJPs after base editing by SaABE or CjABE with each sgRNA. HeLa/CX26 R75W cells were transfected with each base-editing plasmid vector. Negative control cells were left untreated. After transfection for 48 hours, EGFP^+^ cells were sorted, cultured for 1 week, and resorted to collect the EGFP^–^ cells as samples. Samples were seeded into 96-well plates and cultured for 4 days. Samples were then fixed, permeabilized, and incubated with anti-CX26, followed by incubation with Alexa Flour 488–conjugated anti-rabbit IgG and Alexa Flour 555–conjugated cholera toxin subunit B, and then counterstained with DAPI. *n* = 3 technical replicates, representative of 3 independent experiments. Scale bar: 20 μm. (**E–H**) Quantitative length (**E**), area (**F**), form factor (**G**), and LAF (**H**) data presented in **D**. Box-and-whisker plots show median, interquartile range, and minimum and maximum values; isolated dots beyond the whiskers correspond to outliers defined as a value that is smaller than the lower quartile −1.5 × the interquartile range or larger than the upper quartile +1.5 times the interquartile range. Each value was normalized to the value obtained for HeLa/CX26 R75W cells (untreated). *n* = at least 70 analyzed per group. Statistical significance was determined with Kruskal-Wallis test with Dunn’s multiple comparisons test. *P < 0.05, **P < 0.01, ****P < 0.0001.

Our previous study with TG mice carrying the dominant-negative mutation R75W in *GJB2* (CX26*^R75W+^*) revealed fragmentation of GJPs in cochlear cells, and this was also evident in a human cell line overexpressing CX26 R75W; moreover, the R75W mutation negatively affected the accumulation and assembly of gap junction units between cochlear supporting cells (14). Analysis of GJP formation in HeLa cells expressing CX26 WT revealed large and orderly pentagonal/hexagonal plaques on the plasma membrane (Figure 1A). In contrast, the GJPs formed by HeLa cells expressing CX26 R75W showed a drastically fragmented morphology (Figure 1A), resulting in a significant decrease in GJP length compared with that of WT cells (Figure 1B). We first investigated whether ABEs utilizing compact Cas9 orthologs, *Staphylococcus aureus* Cas9 (SaCas9) (22) and *Campylobacter jejuni* Cas9 (CjCas9) (23), could target the R75W mutation. However, SaCas9 and CjCas9 require NNGRRT (where N is any nucleobase and R is A or G) and NNNVRYAC (where V is A, G, or C and Y is T or C) protospacer adjacent motif (PAM) sequences for DNA targeting, making it challenging to target the R75W mutation. Therefore, we used our engineered variants, SaCas9-NNG (our unpublished observations) and enCjCas9 (24), which require NNG and NNNNRYA sequences as their PAMs, respectively. We created expression plasmids encoding SaCas9-NNG D10A or enCjCas9 D8A nickases fused to the TadA8e (referred to as SaABE and CjABE, respectively), the sgRNA and EGFP, and measured base-editing efficiencies toward the CX26 R75W mutation in HeLa cells (Figure 1C). Five sgRNAs (#1 to #5) for SaABE (SaABE#1 to #5), and one sgRNA (#6) for CjABE (CjABE#6), were designed to target the R75W mutation (Figure 1D). The copy number of the *GJB2* R75W mutation in HeLa cells was determined by real-time qPCR, and 3 copies of the transgene were quantified (Supplemental Figure 1; supplemental material available online with this article; https://doi.org/10.1172/jci.insight.185193DS1). These plasmids were transfected into HeLa cells expressing CX26 R75W, and EGFP^+^ cells were sorted 48 hours after transfection (Figure 2A and Supplemental Figure 2A). We then extracted and amplified the *GJB2* R75W transgene (Supplemental Figure 2B) and evaluated the T-to-C (A-to-G) conversions by Sanger sequencing. Sanger sequencing revealed that SaABE exhibited C-to-T conversions at R75W and its surrounding regions, whereas CjABE did not exhibit C-to-T conversions at R75W (Figure 2B). Notably, SaABE#1, #3, and #4 showed high on-target efficiencies, with SaABE#4 exhibiting a higher rate of bystander T-to-C conversions (Figure 2B). Consistent with these results, we observed extensive GJPs in EGFP^+^ cells after transfection of SaABE#1 (Figure 2C).

To further evaluate GJP integrity after base editing, HeLa/CX26 R75W cells were transfected with each base-editing plasmid, and we sorted the EGFP^+^ cells 48 hours after transfection to collect the cells transfected base-editing plasmid. The EGFP^–^ cells were also sorted after culture for 1 week to exclude EGFP^+^ cells, and we directly assessed the effects of base editing (Supplemental Figure 2C). Transfection of SaABE#1 or #3 resulted in the formation of distinct GJPs compared with untreated cells (Figure 2D). Both the length and area of GJPs significantly increased, and their roundness significantly decreased as indicated by the form factor, indicating that the GJPs had elongated along the cell border (Figure 2, E–G). Base editing by SaABE#1 or #3 significantly increased the LAF value (Figure 2H); LAF represents the index for gap junction size and shape by following formula (LAF index = [length rate] [area rate]/[form factor rate]). Therefore, subsequent studies utilized the combination of SaABE with sgRNA#1 or #3 to achieve the highest on-target editing efficiency.

### Optimized base editor enhances on-target editing efficiency and improves the fragmented GJPs.

**Figure 3:**
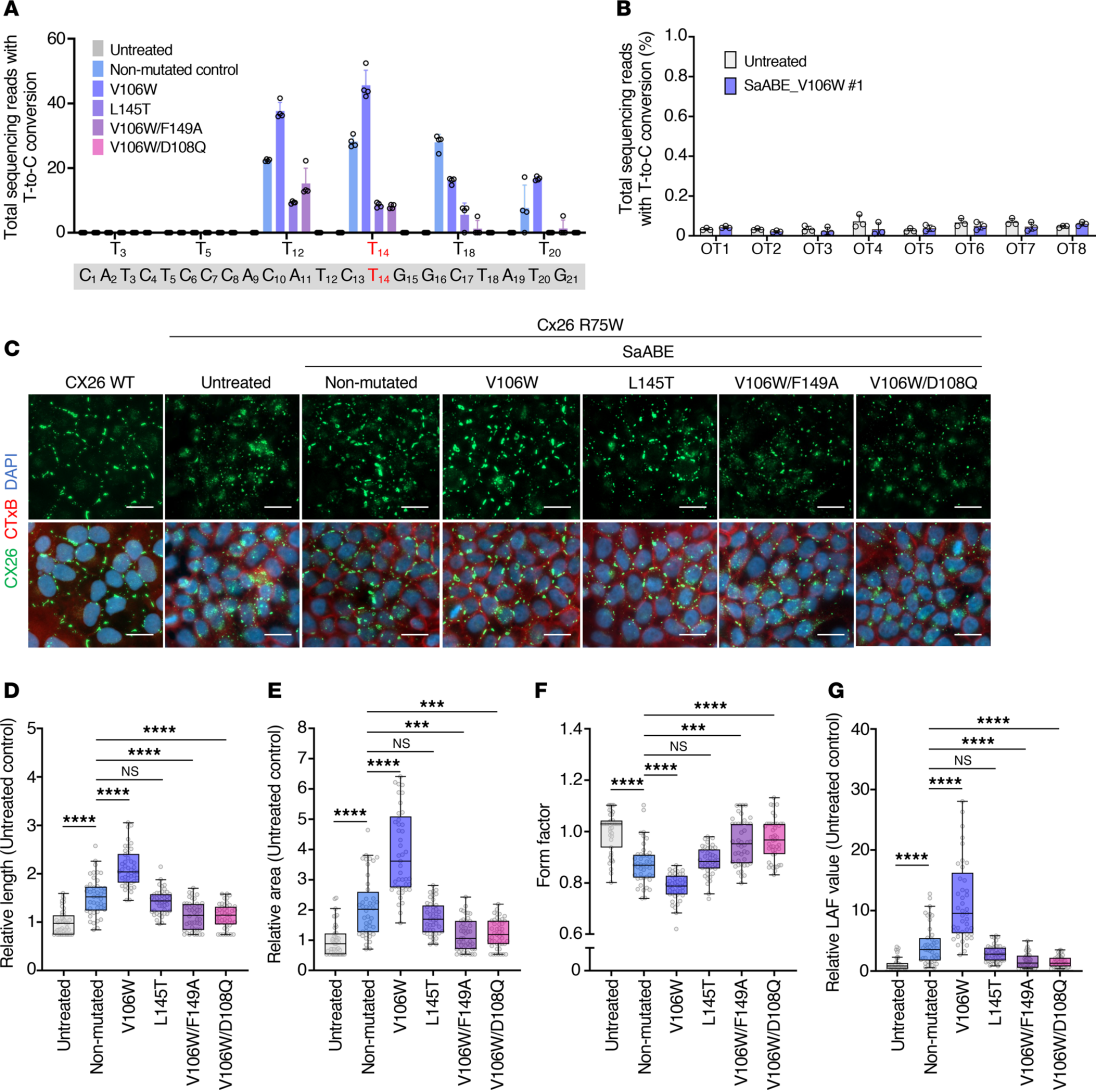
Optimization of base-editing vectors. (**A**) Editing efficiency of the *GJB2* R75W mutation after base editing with each TadA8e mutant. Data represent the mean ± SEM (*n* = 4). (**B**) Amplicon sequencing analysis of the 8 potential off-target sites in *GJB2*. Untreated and base-edited HeLa/CX26 R75W cells after transfection of SaABE_V106W#1. Data represent the mean ± SEM. *n* = 4 per group. (**C**) Representative images of GJPs after base editing by SaABE#1, including TadA8e mutants. HeLa/CX26 R75W cells were transfected with an individual base-editing plasmid. After transfection for 48 hours, EGFP^+^ cells were sorted, cultured for 1 week, and sorted to collect EGFP^–^ cells as samples. Samples were seeded into 96-well plates and cultured for 4 days. Samples were then fixed, permeabilized, and incubated with anti-CX26 antibody, followed by incubation with Alexa Flour 488–conjugated anti-rabbit IgG and Alexa Flour 555–conjugated cholera toxin subunit B, and counterstained with DAPI. *n* = 3 technical replicates, representative of 3 independent experiments. Scale bar: 20 μm. (**D**–**G**) Quantitative length (**D**), area (**E**), form factor (**F**), and LAF (**G**) data presented in **C**. Box-and-whisker plots show median, interquartile range, and minimum and maximum values; isolated dots beyond the whiskers correspond to outliers defined as a value that is smaller than the lower quartile −1.5 × the interquartile range or larger than the upper quartile +1.5 times the interquartile range. Each value was normalized to the value obtained for HeLa/CX26 R75W cells (untreated). *n* = at least 40 GJPs analyzed per group. Statistical significance was determined with Kruskal-Wallis test with Dunn’s multiple comparisons test. ***P < 0.001, ****P < 0.0001.

Recent studies reported that the V106W mutation in TadA8e reduces off-target base editing and bystander effects (25, 26). Incorporating the V106W mutation into SaABE#1 (SaABE_V106W#1) improved its on-target editing efficiency at T_14_ compared with the nonmutated control (Supplemental Figure 3A). Amplicon sequencing revealed that the V106W mutation increased the on-target editing efficiency at T_14_ from 28.2% to 45.6% (Figure 3A). However, the V106W mutation also increased the bystander effect at T_12_ and T_20_ while decreasing it at T_18_ (Figure 3A and Supplemental Figure 3A). Furthermore, SaABE_V106W#1 showed no off-target effect on the human genome (Figure 3B). Consistent with these results, the V106W mutation led to the formation of more extensive and organized GJPs (Figure 3C), significantly increasing both the length and area of GJPs and significantly decreasing the form factor compared with the nonmutated control (Figure 3, D–F). Base editing by SaABE_V106W#1 resulted in a 13.7-fold increase in LAF value compared with the nonmutated control (Figure 3G). These improvements were not observed with sgRNA#3 for unknown reasons (Supplemental Figure 3B and Supplemental Figure 4, A–E). Additionally, other TadA8e mutations reported in previous studies showed no improvement in our experiments (Figure 3G). Therefore, we employed the SaABE_V106W#1–editing vector for the following experiments.

### Base editing recovers the physiological function of GJIC.

**Figure 4:**
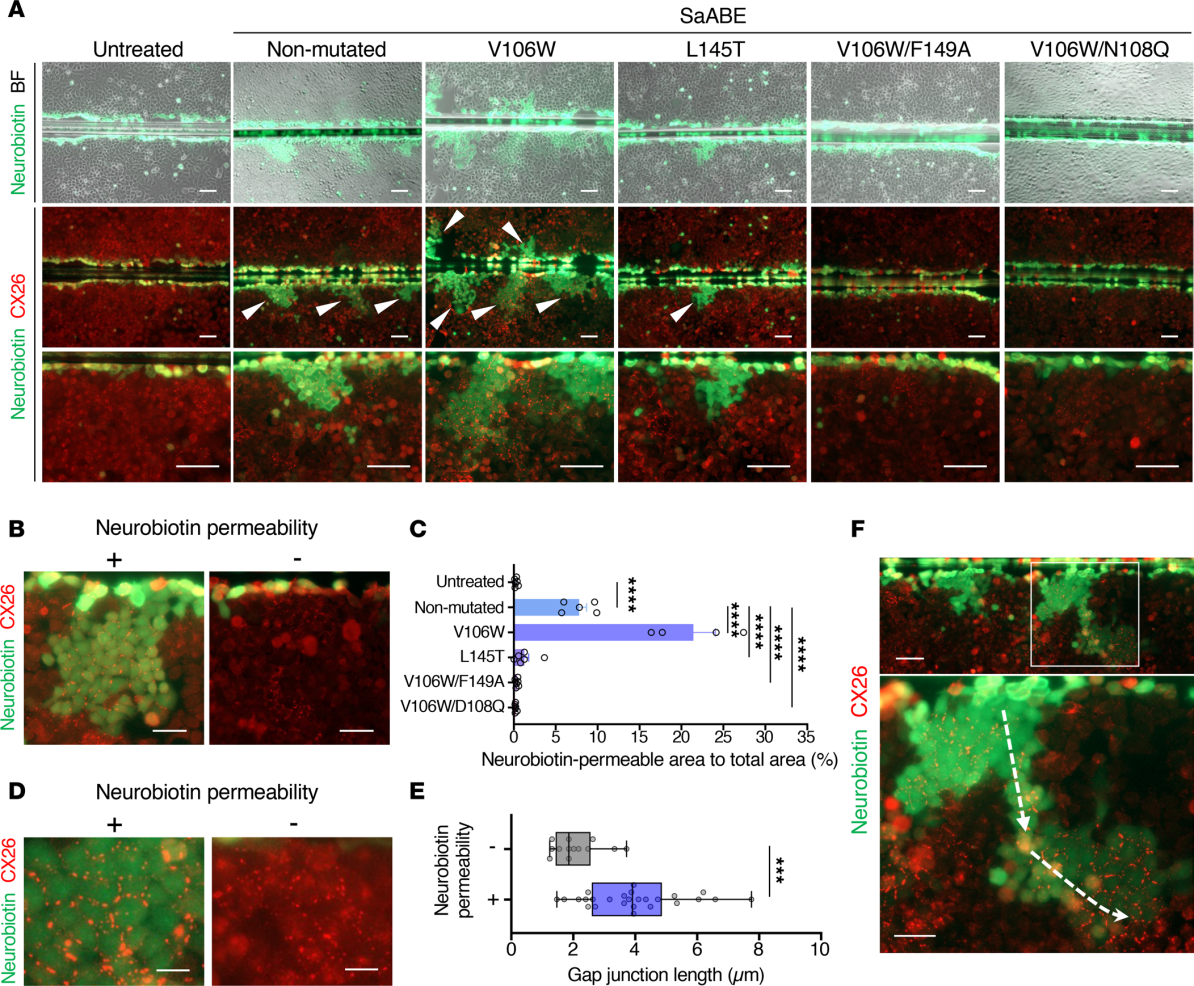
Quantification of gap junction function by intercellular small-molecule permeability. (**A**) To quantify permeability of small molecules through gap junctions after base editing, monolayers of base-edited HeLa/CX26 R75W cells were scrape-loaded with Neurobiotin (green) and stained with anti-CX26 antibody (red). Arrowheads indicate sites of Neurobiotin permeability. *n* = 3 technical replicates, representative of 3 independent experiments. Scale bar: 100 μm. (**B**) Representative images of the area permeable to Neurobiotin (left, +) or nonpermeable (right, –) after base editing. (**C**) Quantification of data presented in **B**. The area of cells that were permeable to Neurobiotin after wounding was quantified. Each value represents the mean ± SEM. *n* = at least 4 per group. Statistical significance was determined with Kruskal-Wallis test with Dunn’s multiple comparisons test. ****P < 0.0001. Scale bar: 50 μm. (**D**) Representative images of GJPs that were permeable to Neurobiotin (left panel, +) or non permeable (right, –) after base editing. (**E**) Quantification of data presented in **D**. The length of each GJP was quantified. The box-and-whisker plot shows median, interquartile range, and minimum and maximum values; isolated dots beyond the whiskers correspond to outliers defined as a value that is smaller than the lower quartile −1.5 × the interquartile range or larger than the upper quartile +1.5 times the interquartile range. *n* = at least 13 analyzed per group. Statistical significance was determined with the Mann-Whitney *U* test. ***P < 0.001. Scale bar: 10 μm. (**F**) Representative images of Neurobiotin permeability to more distant area. Arrow shows the flow of Neurobiotin. Scale bar: 100 μm.

GJPs contribute to the homeostasis of the ionic environment of the inner ear by transporting K^+^ between inner ear cells (13). To evaluate the physiological function of GJPs after base editing, we utilized the neuronal tracer Neurobiotin, which passes through the GJIC network in the same way as K^+^ and other ions. The area of GJIC-mediated Neurobiotin permeability was quantified by scrape loading and dye transfer assays on confluent cell monolayers of base-edited cells. Neurobiotin permeability was not observed in HeLa/CX26 R75W cells (Figure 4A, untreated). In contrast, consistent with high-base-editing efficacy (Figure 3A) and repair of GJPs (Figure 3G), Neurobiotin permeability was frequently observed in V106W mutation (Figure 4A, arrowheads). The treatment with base-editing construct with V106W mutation in ABE8e drastically increased its permeability compared with the nonmutated control (Figure 4, B and C). Importantly, in SaABE_V106W#1, the length of GJPs in the permeabilized region was significantly increased compared with the length where no permeabilization was observed (Figure 4, D and E). These results imply that repair of GJPs resulted in restoration of Neurobiotin permeability, that is, recover of the physiological function of GJIC. Neurobiotin was also permeated to more distant area through the partially repaired GJP colonies (Figure 4F, arrows shows the flow of Neurobiotin). Based on these results, we finally selected the SaABE_V106W#1 construct to carry out base editing for *GJB2* R75W treatment.

### AAV-mediated base editing improves GJP formation and recovers GJIC.

**Figure 5:**
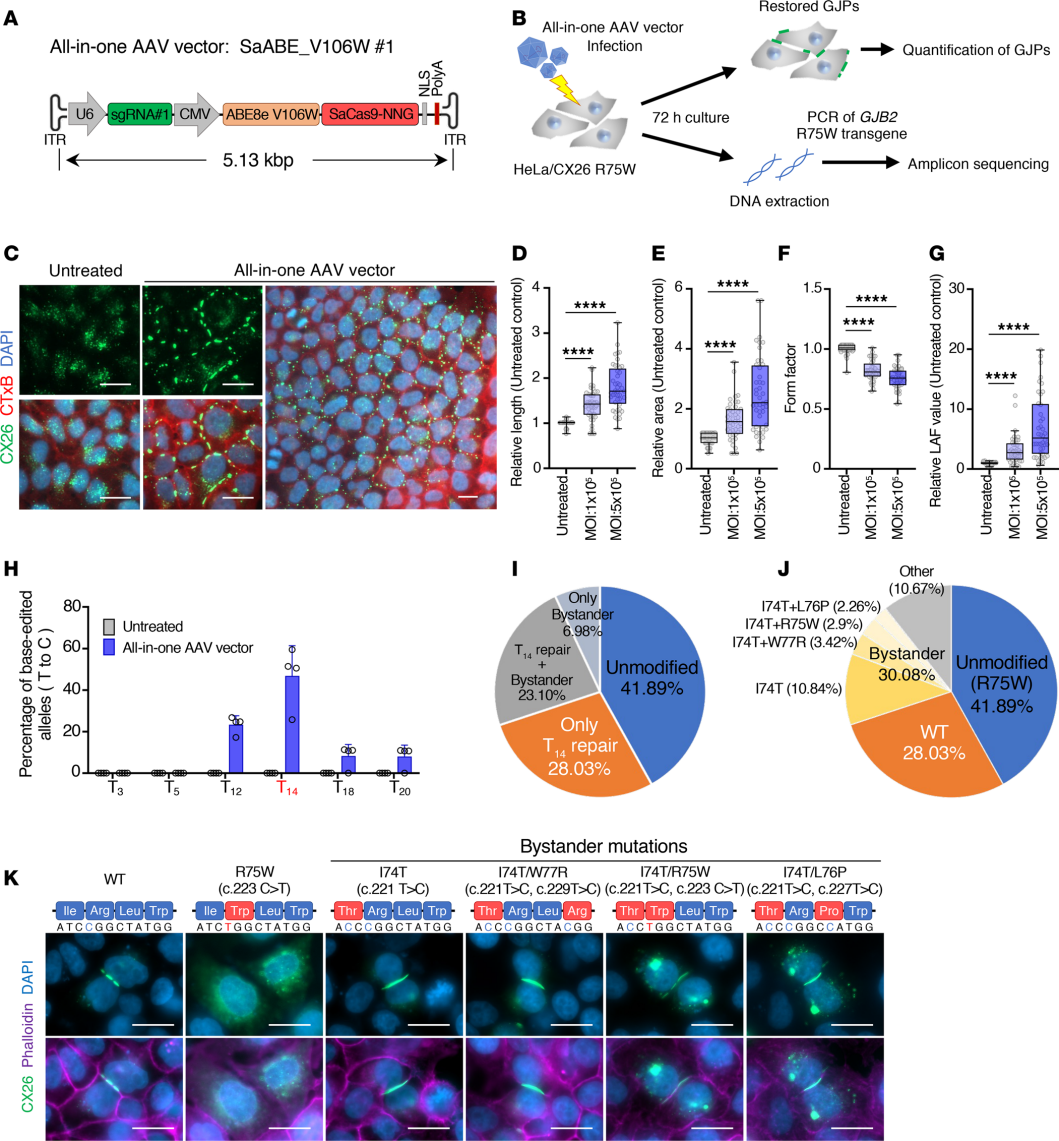
Restoration of GJPs by AAV-mediated base editing. (**A**) Schematic diagram of the genome of the all-in-one AAV vector encoding SaABE_V106W#1. (**B**) Strategy for assessing the efficiency of AAV-mediated base editing at *GJB2* R75W. (**C**) Representative images of GJPs after all-in-one AAV-mediated base editing. HeLa/CX26 R75W cells were infected with the all-in-one AAV vector. After transfection for 72 hours, cells were fixed, permeabilized, and incubated with anti-CX26 antibody, followed by incubation with Alexa Flour 488–conjugated anti-rabbit IgG and Alexa Flour 555–conjugated cholera toxin subunit B, and then counterstained with DAPI. Scale bar: 10 μm. (**D**–**G**) Quantitative length (**D**), area (**E**), form factor (**F**), and LAF (**G**) data after all-in-one AAV-mediated base editing (MOI of 1 × 10^5^ or 5 × 10^5^ vg/cell). Box-and-whisker plots show median, interquartile range, and minimum and maximum values; isolated dots beyond the whiskers correspond to outliers defined as a value that is smaller than the lower quartile −1.5 × the interquartile range or larger than the upper quartile +1.5 times the interquartile range. Each value was normalized to the value obtained for HeLa/CX26 R75W cells (untreated). *n* = 44 analyzed per group. Statistical significance was determined with Kruskal-Wallis test with Dunn’s multiple comparisons test. ****P < 0.0001. (**H**) Efficiency of correction of the mutation *GJB2* R75W in HeLa cells after infection with the all-in-one AAV vector. Data represent the mean ± SEM. *n* = 4. (**I**) Pie charts showing the composition of allelic variants by on-target or bystander editing after AAV-mediated base editing. (**J**) Pie charts showing the composition of allelic variants after AAV-mediated base editing. (**K**) Representative images of GJPs generated by bystander effect. Scale bar: 10 μm.

We evaluated AAV-mediated therapeutic efficacy by our selected base-editing vector. Although our selected cassette is 5.13 kbp, which exceeds the recommended 4.7 kbp for AAV packaging, there are some cases where packaging of less than 5.2 kbp has been successfully achieved (21). Therefore, we first confirmed AAV-mediated base-editing ability using HeLa/CX26 R75W cells. Our previously generated vector, AAV-Sia6e, which efficiently infects cochlear supporting cells in the inner ear (our unpublished observations), showed high infectious tropism to HeLa cells (Supplemental Figure 5). We developed an all-in-one AAV vector, which encoded all the component of base editing (SaABE_V106W#1) (Figure 5A), and evaluated GJP integrity after base editing (Figure 5B). Infection with the all-in-one AAV vector resulted in more extensive formation of GJPs between cells compared with untreated cells (Figure 5C), and both the length and area of GJPs significantly increased and the form factor significantly decreased (Figure 5, D–F). Moreover, LAF value increased approximately 3- to 7-fold compared with the untreated group, with dose dependency (Figure 5G). Next, we evaluated base-editing efficiency and specificity by the all-in-one AAV vector. On-target T-to-C conversion at the target (T_14_) was 46.8%, with some bystander editing at T_12_, T_18_, and T_20_ (Figure 5H), comparable to 45.6% of base editing by EGFP sorting after plasmid transfection (Figure 3A). These results demonstrated that, although our base editing cassette slightly exceeds the recommended genome size for AAV packaging, the all-in-one AAV vector was functional. The rates of T_14_-only repair, T_14_ and bystander repair, bystander-only repair, and unmodified repair allelic variants after AAV-mediated base editing were 28.03%, 23.10%, 6.98%, and 41.89%, respectively (Figure 5I); AAV-mediated base editing restored 28.03% to WT sequence (equal to the base-editing rate of T_14_-only repair [28.03%] in Figure 5I), and bystander editing occurred at a rate of 30.08% (equal to the base-editing rate of T_14_ repair and bystander [23.10%] and bystander only [6.98%] in Figure 5I) (Figure 5J). Transient expression of the most frequent bystander mutations in HeLa cells showed that I74T (most frequent) and I74T/W77R (second-most frequent) formed GJPs similar to WT, and I74T/R75W (third-most frequent) and I74T/L76P (fourth-most frequent) also formed GJPs similar to WT with some fragmented plaques in the cytoplasm and nucleus (Figure 5, J and K). Therefore, it is considered that the drastically fragmented morphology of GJPs caused by R75W was repaired in 42.29% of allelic variants after AAV-mediated base editing, including T14-only repair (28.03%), I74T repair (10.84%), and I74T+W77R repair (3.42%) (Figure 5J).

**Figure 6:**
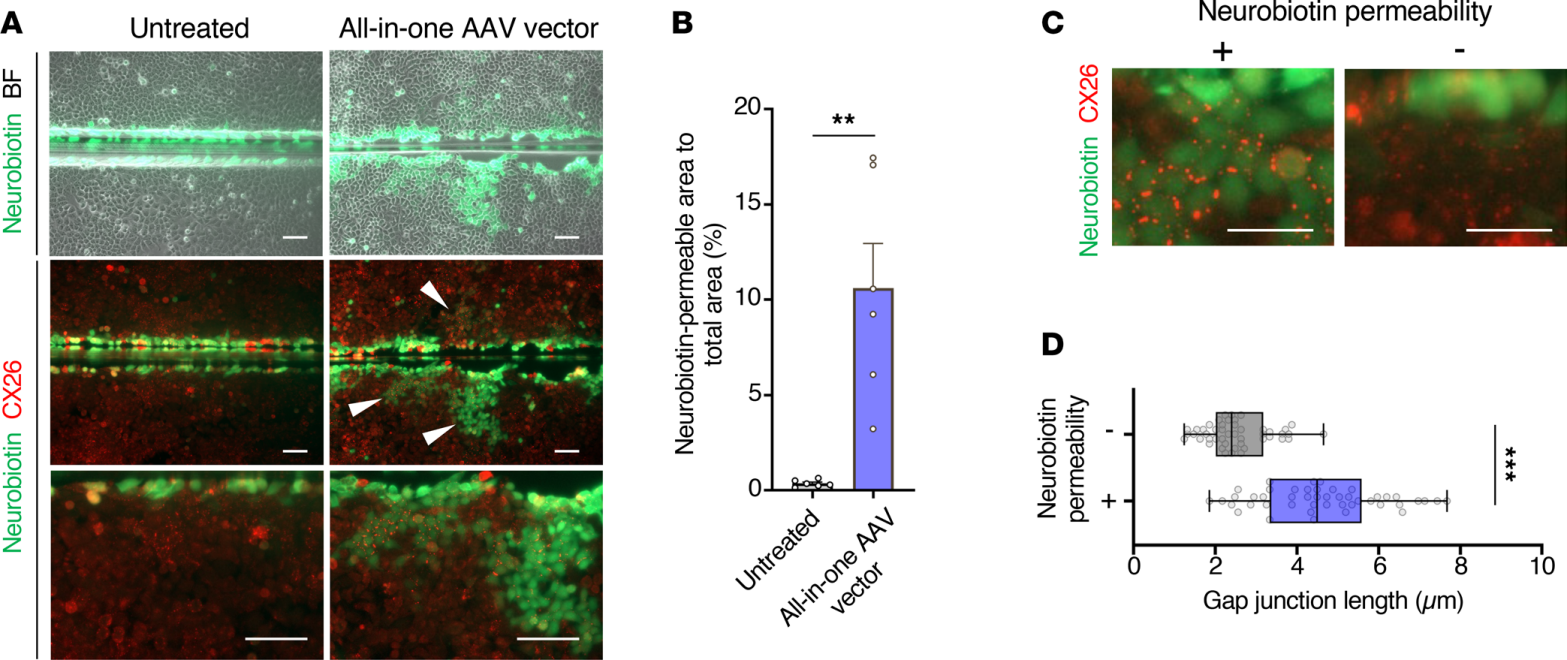
Recovery of physiological function of gap junctions after all-in-one AAV-mediated base editing. (**A**) Representative images of the assessment of Neurobiotin permeability. To quantify permeability of small molecules through gap junctions after infection with the all-in-one AAV vector, monolayers of base-edited HeLa/CX26 R75W cells were scrape-loaded with Neurobiotin (green) and stained with anti-CX26 antibody (red). Arrowheads indicate sites of Neurobiotin permeability. Scale bar: 100 μm. (**B**) Quantification of data presented in **A**. The area of cells that were permeable to Neurobiotin after wounding was quantified. Each value represents the mean ± SEM. *n* = 6 per group. Statistical significance was determined with the Mann-Whitney *U* test. **P < 0.01. (**C**) Representative images of GJPs with Neurobiotin permeability (left, +) or nonpermeability (right, –) after base editing. (**D**) The length of each GJP was quantified. Box-and-whisker plot shows median, interquartile range, and minimum and maximum values; isolated dots beyond the whiskers correspond to outliers defined as a value that is smaller than the lower quartile −1.5 × the interquartile range or larger than the upper quartile +1.5 times the interquartile range. *n* = 45 per group. Statistical significance was determined with the Mann-Whitney *U* test. ***P < 0.001. Scale bar: 10 μm.

To evaluate GJIC after AAV-mediated base editing, HeLa/CX26 R75W cells were infected with the all-in-one AAV vector and analyzed for permeability by Neurobiotin. The GJPs formed by the base-edited cells had a wider range of permeability than the untreated cells (Figure 6A), and the permeability ratio was significantly increased by infection with the all-in-one AAV vector (Figure 6B). The length of GJPs in Neurobiotin-permeable regions was significantly greater than that measured in nonpermeable regions (Figure 6, C and D).

### AAV-mediated base editing restores the formation of normal GJPs of inner sulcus cells in dominant-negative CX26 R75W TG mice.

**Figure 7:**
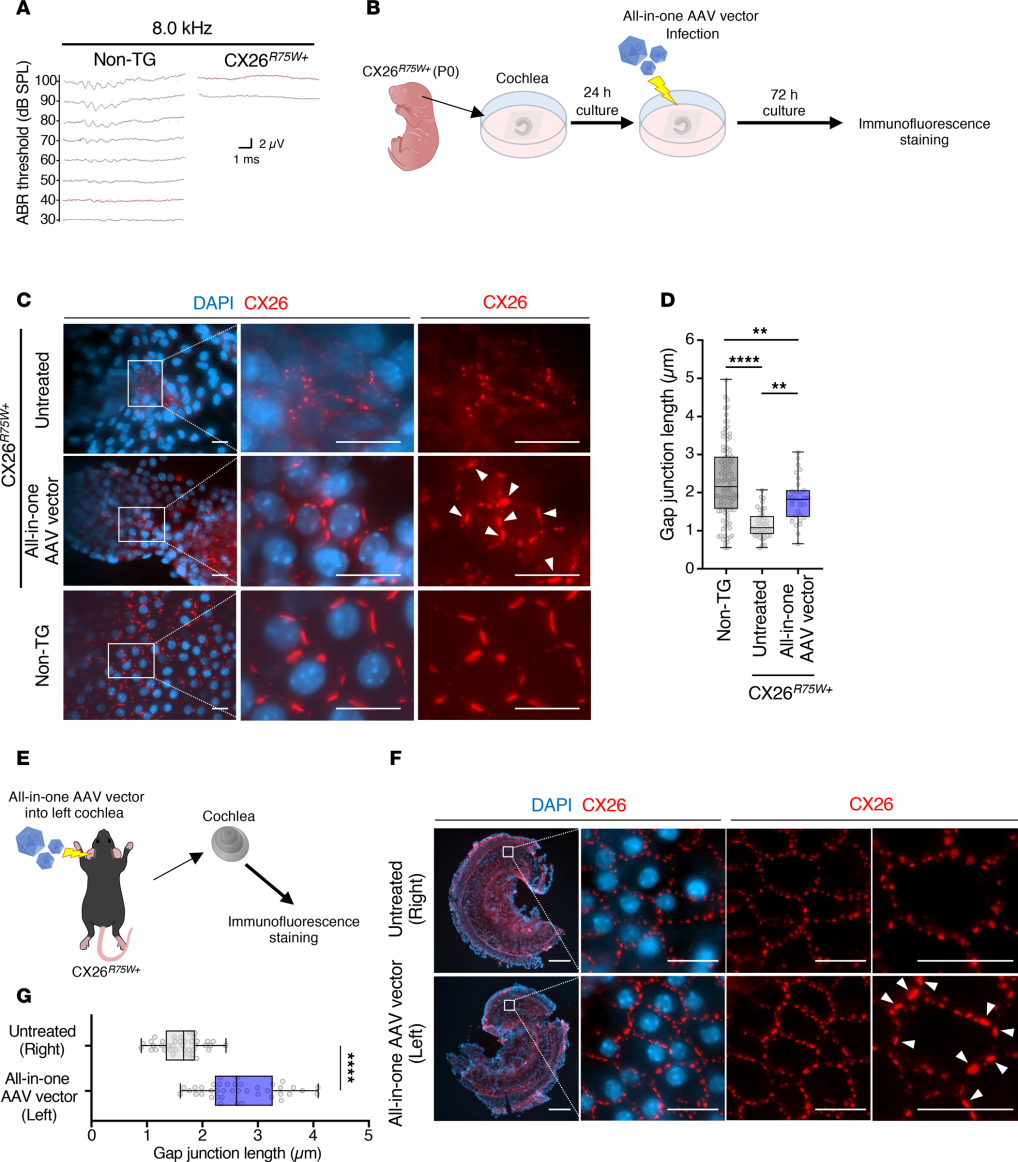
All-in-one AAV-mediated base editing in transgenic mice with GJB2 R75W. (**A**) Auditory brain stem response (ABR) waveforms of *GJB2* R75W–transgenic mice (CX26*^R75W+^*) and their nontransgenic littermates (non-TG) at 30 weeks of age (*n* = 3). The number to the left side of each wave indicates sound pressure level (SPL) expressed in decibels (dB). (**B**) Strategy for assessing the efficiency of AAV-mediated base editing of CX26*^R75W+^* mice. (**C**) Representative images of cochlea after all-in-one AAV-mediated base editing. Cochlea organotypic cultures were prepared from P0 CX26*^R75W+^* mice and non-TG littermates (*n* = 3). Cochlea samples were infected with the all-in-one AAV vector and maintained in DMEM for 72 hours. Samples were then fixed, permeabilized, and incubated with anti-CX26 antibody, followed by incubation with Cy3-conjugated anti-rabbit IgG, and then counterstained with DAPI. Arrowheads indicate distinct GJPs after AAV-mediated base editing. Scale bar: 10 μm. (**D**) Quantification of data presented in **C**. Length of GJPs from each group of inner sulcus cells. Box-and-whisker plot shows median, interquartile range, and minimum and maximum values; isolated dots beyond the whiskers correspond to outliers defined as a value that is smaller than the lower quartile −1.5 × the interquartile range or larger than the upper quartile +1.5 times the interquartile range. *n* = at least 42 per group. Statistical significance was determined with Kruskal-Wallis test with Dunn’s multiple comparisons test. **P < 0.01, ****P < 0.0001. (**E**) The all-in-one AAV vector was injected into the cochlear perilymph of left ear via the semicircular canal of adult CX26*^R75W+^* mice (*n* = 3). After administration of the AAV vector, the length of gap junction in cochlea tissue was evaluated. (**F**) Representative images of cochlea after AAV-mediated base editing. Cochlea samples were fixed, permeabilized, and incubated with anti-CX26 antibody, followed by incubation with Cy3-conjugated anti-rabbit IgG, and then counterstained with DAPI. Arrowheads indicate distinct GJPs after AAV-mediated base editing. Scale bar: 200 μm (leftmost images), 20 μm (images in other columns). (**G**) Quantification of data presented in **F**. Length of GJPs of inner sulcus cells was evaluated. The explanation of the box-and-whisker plot is the same as in **D**. *n* = 40 per group. Statistical significance was determined with Kruskal-Wallis test with Dunn’s multiple comparisons test. ****P < 0.0001.

TG mice expressing human CX26 R75W (CX26*^R75W+^* mice) show severe hearing loss, as is also observed in humans (27). Therefore, we evaluated the therapeutic efficacy of an all-in-one AAV vector using cochlear tissue from TG mice. Hearing was tested for both TG and non-TG mice at the same week of age, revealing severe hearing loss only in the TG mice (Figure 7A). Cochlear organotypic cultures from newborn TG mice (P0) were collected and infected with the all-in-one AAV vector (Figure 7B). The inner sulcus cells in the untreated group had drastically fragmented and small vesicle–like GJPs (Figure 7C). In contrast, the inner sulcus cells infected with the all-in-one AAV vector formed distinct GJPs with orderly pentagonal or hexagonal structures around cells that were similar to those of WT cells (Figure 7C), and the length of GJPs was significantly increased compared with results obtained for untreated cochlea (Figure 7D). The AAV vector was also locally injected into the cochlea perilymph via the semicircular canal in adult CX26*^R75W+^* mice (Figure 7E). The inner sulcus cells in the untreated side (right ear) had drastically fragmented and small vesicle–like GJPs (Figure 7F). In contrast, the inner sulcus cells infected with the all-in-one AAV vector (left ear) formed large and planar GJPs (Figure 7F), and the length of GJPs was significantly increased compared with results obtained for untreated side (Figure 7G). These results revealed that the AAV-mediated base editing effectively restores the structure of GJPs in inner sulcus cells, suggesting a potential therapeutic approach for severe hearing loss caused by the CX26 R75W mutation.

## Discussion

Recently, the world’s first clinical trial of AAV-mediated gene therapy for *OTOF*, which causes severe hearing loss, was conducted in the United States and China, and favorable therapeutic effects were reported (National Clinical Trial no. NCT05788536 in US and Chinese Clinical Trial Register no. ChiCTR2200063181 in China). In recent months, several groups also have reported on AAV gene therapy targeting *OTOF* (28–30), and AAV-mediated gene therapy for hearing loss is becoming a reality.

*GJB2*-related hearing loss is the most common type of genetic deafness worldwide, yet no drug or *GJB2*-targeted treatment is available. We previously reported that CX26 regulates the accumulation of gap junction components and the assembly of gap junctions in the cell-cell junctions between cochlear supporting cells and that mutation of *GJB2* leads to fragmentation of GJPs, resulting in hearing loss (14). As a novel therapeutic approach for *GJB2*-related hearing loss, we recently developed AAV-Sia6e, which is a vector suitable for treating *GJB2*-related hearing loss with high infectivity of Deiters’ cells, Hensen’s cells, inner and outer sulcus cells, and fibrocytes of the inner ear and reported that this AAV-mediated *Gjb2* gene transfer restored the GJIC network and rescued hearing in a *Gjb2* conditional knockout mouse model (our unpublished observations). Based on the aforementioned reports, gene-replacement therapy can be an effective option for treatment of genetic deafness related to decreased expression and function of CX26, such as recessive inherited hearing loss. In contrast, gene-replacement therapy is not effective for the treatment of dominant-negative mutations, including *GJB2* R75W, which cause genetic deafness via suppression of the normal function of CX26. The long-term treatment of this disease does not require gene-replacement therapy or transient nucleic acid drug therapy such as siRNA or an antisense oligonucleotide but rather treatment that corrects the underlying genomic mutation(s).

ABE repairs only a single mutated site within a genome. Therefore, it has the advantage over conventional CRISPR/Cas genome editing with donor DNA because ABEs do not lead to undesirable effects such as insertions or deletions. To date, both nonviral and viral delivery methods have shown great promise for delivering ABEs for in vivo therapeutic purposes in rodents and primates (31, 32). In vivo delivery through dual AAV vector using a split-intein delivery system has achieved efficient editing in a wide range of tissues and cell types, including sensory hair cells of the inner ear (33). Moreover, a single AAV vector system that allows local replacement of a mutated sequence with its WT counterpart, based on combined CRISPR/Cas9 and microhomology-mediated end joining (MMEJ), results in superior therapeutic efficacy (34). However, in general, the recommended genome size that can be loaded onto an AAV is ≤4.7 kb, and although it is possible to load larger genomes, albeit inefficiently, it has been reported that ≤5.2 kb is the genome size limit (21, 35). The widely used ABE8e using SpCas9 (4.1 kbp) greatly exceeds the genome size that can be loaded on AAV. On the other hand, compact CRISPR/Cas enzymes, such as SaCas9 (3.2 kbp) or enAsCas12f (1.3 kbp) (36) requires relatively long PAM sequences for DNA targeting, restricting their application. In this study, we developed the base-editing cassette SaABE that contains all the components required for genome editing and has looser PAM restrictions than conventional SaCas9. Although our base-editing cassette (5.13 kbp) was slightly larger than the recommended size for AAV, it was successfully loaded onto the AAV-Sia6e vector using standard synthesis methods, suggesting that other AAV serotypes may also be loaded with this cassette. An all-in-one AAV vector carrying this base-editing cassette on AAV-Sia6e also showed high base-editing efficiency. Therefore, our developed base-editing cassette would be the current best ABE for the treatment of *GJB2* R75W mutation. The base-editing vector consisting of SaABE_V106W#1 drastically restored the permeability of GJPs (Figure 4C), and Neurobiotin permeability was observed in colonies forming long and condensed GJPs (Figure 4, D and E). These results indicate that the changes in membrane localization of CX26 associated with repair of the *GJB2* gene mutation restore the substance permeability of GJPs, that is, the physiological function such as ion permeability. Moreover, the partially repaired GJP colonies were connected to each other in one part and Neurobiotin permeated extensively through this part (Figure 4F). Thus, even though not all the abnormal GJPs in cochlear supporting cells were restored to normal functionality, it was possible to improve the ionic environment in the inner ear by partially connecting colonies with repaired GJPs and forming a path for K^+^ recycling. Additionally, since our results represent the effect of genome-editing therapy on cells with 3 copies of *GJB2* R75W (HeLa cells expressing *GJB2* R75W), it can be expected that the editing effect will be even greater on normal diploid cells.

AAV-mediated base editing repaired the fragmented GJPs in cochlear tissue from both perinatally and mature *CX26^R75W+^* TG mice after only a single administration (Figure 7, C, D, F, and G) and further increased the repair efficiency in a dose-dependent manner (Figure 5G). The fact that the inner ear is embedded in the skull makes it difficult to administer multiple doses. Therefore, these results indicate that it is possible to treat *GJB2*-derived genetic deafness with a single therapeutic dose by adjusting the concentration of the administered AAV vector. Our results showed that AAV-mediated base editing to the dominant-negative mutation in *GJB2* enabled histologic improvement of the fragmented GJPs in inner sulcus cells, which is not possible with gene-replacement therapy. Larger and longer experiments are required to assess improvement of hearing loss and currently evaluating the therapeutic efficacy to auditory function by AAV-mediated base editing. We are also investigating the clinical application of this AAV-mediated base-editing therapy by working with induced pluripotent stem cells derived from Japanese patients with the *GJB2* R75W mutation. Our constructed ABE cassette can also be used for the treatment of large genes that cannot be loaded into an AAV, such as *CDH23*, which is the causative gene of juvenile-onset bilateral sensorineural hearing loss, and Usher syndrome–related genes, by recombining the sgRNA of ABE. For such applications, our ABEs can be loaded into AAVs such as Anc80L65, which are highly infectious for inner ear hair cells, to enable genome-editing therapy for hair cells.

While ABEs have the advantage of editing only the mutation site, they have the problem of repairing the neighboring bases when multiple identical bases exist near the target site, such as T_12_, T_18_, and T_20_ near the on-target T_14_. Among the bystander mutations, I74T and L76P have not been reported to be pathogenic, and they formed GJPs similar to WT. Although W77R is reported to be pathogenic (37), its combination with I74T (I74T/W77R) formed GJPs similar to WT. Surprisingly, linear GJPs were also observed on the cell membrane even in I74T/R75W, in which R75W remains unedited (Figure 5K). These results imply that I74T mutation may suppress the pathogenicity of R75W and W77R, suggesting that the major bystander mutations that occurred in this study do not affect the formation of GJPs. Nonetheless, the development of more mutation-specific base-editing cassettes packageable into an all-in-one AAV vector is required to enhance the precision and therapeutic potential of base editing for hearing loss caused by *GJB2* mutations and other genetic diseases.

In this study, we constructed a small ABE that can be packaged into AAV vector to repair the pathogenic mutation in *GJB2* and revealed that AAV-mediated base editing is enabled to repair the abnormal GJPs produced as a consequence of a dominant-negative *GJB2* mutation and restore the physiological function of GJPs. Our results suggest a therapeutic opportunity for AAV-mediated base editing for the treatment of dominant-negative *GJB2*-related hearing loss or skin diseases not possible with gene-replacement therapy. We anticipate that an all-in-one AAV vector — by virtue of its compact size and broad targeting range — will enable a range of therapeutic applications for a variety of hereditary deafness with improved safety and efficacy owing in part to ABE packaging into a single-vector system.

## Methods

### Sex as a biological variable.

Our study examined male and female animals, and similar findings are reported for both sexes.

### Animals.

Dominant-negative CX26 R75W (CX26*^R75W+^*) TG mice were obtained from a breeding colony of a previously reported line (27). These TG mice were maintained on a C57BL/6J background and crossed with C57BL/6J mice to generate R75W TG offspring. Non-TG littermates with a C57BL/6J background were used as controls.

### Cell lines.

HeLa cell lines stably expressing CX26 WT and CX26 R75W were provided by Sabrina Yum (Children’s Hospital of Philadelphia, Philadelphia, Pennsylvania, USA). Briefly, human *GJB2* ORF was obtained by reverse transcription-PCR (Superscript II; Life Technologies, 18064022) and subcloned into vector pIRESpuro3, then the R75W mutation was introduced into the ORF of human *GJB2* cDNA by PCR site–directed mutagenesis using the QuikChange kit (Agilent, 200518). HeLa cell lines were stably transfected with plasmids containing WT human CX26 (HeLa/CX26 WT) and R75W mutant (HeLa/CX26 R75W) using Lipofectamine 2000 (Invitrogen, 11668027). The cells were cultured in DMEM (WAKO, 041-29775) containing 0.5 μg/mL puromycin and 10% FBS, and single colonies were established (38).

### Construction of base-editing plasmid vector.

The two base-editing vectors used to edit the mutation in this study, namely SaCas9-NNG-ABE8e (SaABE) and enCjCas9-ABE8e (CjABE), were constructed by VectorBuilder. All sgRNA (#1 to #6) targeting the T-to-C conversion at R75W in *GJB2* were designed using an online webtool (Benchling, https://www.benchling.com/) (Figure 1D). The vector IDs are as follows: VB220620-1040dnx (SaABE#1), VB221024-1475wfy (SaABE#2), VB221024-1477xed (SaABE#3), VB221024-1478jxf (SaABE#4), VB221024-1479afb (SaABE#5), and VB220620-1054pua (CjABE#6), which can be used to retrieve detailed information about each vector at https://en.vectorbuilder.com/

### Sanger sequencing.

HeLa/CX26 R75W cells were maintained in DMEM supplemented with 10% FBS and cultured at 37°C with 5% CO_2_. The cells were seeded into 12-well plates at 1 × 10^5^ cells per well at 48 hours before transfection. The cells were transfected with base-editing vectors using FuGENE HD (Promega, E2311). After transfection for 48 hours, EGFP^+^ cells were sorted using a BD FACSAria III cell sorter (Becton Dickinson), and genomic DNA was isolated using a DNeasy Blood & Tissue kit (Qiagen, 69504). The *GJB2* R75W transgene was amplified using PrimeSTAR HS (Takara, R010A) and the following primer set: forward, 5′-CGCAAATGGGCGGTAGGCGTG-3′, and reverse, 5′-CTTCTCATGTCTCCGGTAGG-3′. PCR cycling was as follows: initial denaturation for 5 minutes at 94°C, followed by 40 cycles of 10 seconds at 98°C, 5 seconds at 58°C, and 60 seconds at 72°C, with final extension for 5 minutes at 72°C. The PCR product was purified using a FastGene Gel/PCR extraction kit (NIPPON Genetics Co., FG-91202). Sanger sequencing was performed by Eurofins Genomics.

### Amplicon sequencing.

The same genomic DNA used for Sanger sequencing was subjected to amplicon sequencing conducted by Seibutsu Giken using the MiSeq platform (Illumina). A primer set (forward: 5′-CGCAAATGGGCGGTAGGCGTG + adapter sequence-3′, reverse: 5′-CTTCTCATGTCTCCGGTAGG + adapter sequence-3′) was designed to amplify the *GJB2* R75W transgene. The base-editing outcomes, including off-target editing, were determined using CRISPResso2 software. The in silico prediction algorithm CRISPOR was used for off-target analysis.

### Immunofluorescence staining.

Cells were fixed with 4% paraformaldehyde (PFA) and then permeabilized with 0.1% Triton X-100 (WAKO, 169-21105) in PBS. Samples were blocked with 2% BSA in PBS and incubated with an antibody specific for CX26 (1:600, Invitrogen, 71-0500), followed by incubation with Alexa Flour 488–conjugated anti-rabbit IgG (1:1,000, Invitrogen, A11008) or Cy3-conjugated anti-rabbit IgG (1:1,000, Invitrogen, A10520) and Alexa Fluor 633–conjugated phalloidin (1:400, Invitrogen, A22284) or Alexa Fluor 488–conjugated phalloidin (1:400, Invitrogen, A12379) and counterstained with DAPI (Vector Laboratories, H-1200). Fluorescence images were captured with an Observer.Z1 microscope (Carl Zeiss), and fluorescence intensity was calculated with software from Keyence.

### Quantification of base-edited GJPs.

HeLa/CX26 R75W cells were transfected with an individual base-editing vector. After transfection for 48 hours, EGFP^+^ cells were sorted, cultured for 1 week, and resorted to collect the EGFP^–^ cells as samples. Cells were seeded into Lumox multiwell 96-well plates (Sarstedt, 94.6000.024) and cultured at 37°C with 5% CO_2_ for 4 days. Cells were fixed with 4% PFA and then permeabilized with 0.1% Triton X-100 in PBS. Samples were blocked with 2% BSA in PBS and incubated with anti-CX26 antibody (1:600, Invitrogen, 71-0500) followed by incubation with Alexa Flour 488–conjugated anti-rabbit IgG (1:400, Invitrogen, A12379) and Alexa Flour 555–conjugated cholera toxin subunit B (1:300, Invitrogen, C34776) and then counterstained with DAPI (1:2,000, Dojindo, SB033). Fluorescence images were acquired with the IN Cell Analyzer 2200 Cell Imaging system (GE Healthcare Life Sciences), and fluorescence was quantified using IN Cell Developer toolbox software v1.9 (GE Healthcare Life Sciences). The diameter, area, and roundness of GJPs bordering the 1-cell region, as indicated by the form factor, were measured, and the GJPs having the overall greatest diameter, largest area, and least roundness were extracted. The LAF value (LAF index) was defined as the value calculated by the following formula: LAF index = [length rate] [area rate]/[form factor rate], where form factor rate is a measure of roundness.

### Scrape loading and dye transfer assay.

To quantify GJIC after base editing, monolayers of confluent cells were washed with HBSS (Invitrogen, 14025-092) and then immersed in Neurobiotin tracer (Vectorlabs, SP-1120). Each monolayer was then wounded with a scalpel; then, for 5 minutes, wounded cells were allowed to absorb Neurobiotin and exchange Neurobiotin between cells through gap junctions. Samples were then washed in HBSS, fixed with 4% PFA in PBS, and permeabilized in PBS containing 0.1% Triton X-100. Samples were then blocked with 2% BSA in PBS and incubated with anti-CX26 antibody (1:600, Invitrogen, 71-0500) followed by incubation with Cy3-conjugated anti-rabbit IgG (1:1,000, Invitrogen, A10520) and Alexa Flour 488–conjugated streptavidin (1:1,000, Invitrogen, S11223) and then counterstained with DAPI (Vector Laboratories, H-1200). Fluorescence images were captured with a Zeiss Observer.Z1 microscope (Carl Zeiss), and Neurobiotin permeability was quantified by counting the area of cell layers that received Neurobiotin from the wounded cells via gap junctions using Keyence software.

### Construction of an all-in-one AAV vector.

The all-in-one AAV vector was constructed and packaged by VectorBuilder. The vector ID is VB230407-1007xrm, which can be used to retrieve detailed information about the vector on https://en.vectorbuilder.com/

### Analysis of base-editing efficacy via the all-in-one AAV vector.

HeLa/CX26 R75W cells were infected with the all-in-one AAV vector at a multiplicity of infection (MOI) of 1 × 10^6^ viral genomes (vg) per cell and maintained in DMEM containing 10% FBS at 37°C and 5% CO_2_. After incubation for 72 hours, the infected cells were harvested and subjected to amplicon sequencing as described in *Sanger sequencing* and *Amplicon sequencing*.

### Quantification of the AAV-mediated base-edited GJPs.

HeLa/CX26 R75W cells were seeded into Lumox multiwell 96-well plates (Sarstedt, 94.6000.024) and cultured at 37°C with 5% CO_2_ for 24 hours. Cells were then infected with the all-in-one AAV vector at a MOI of 1 × 10^5^ vg/cell or 5 × 10^5^ vg/cell and maintained in DMEM containing 10% FBS. After incubation for 72 hours, the infected cells were stained with anti-CX26 antibody (1:600, Invitrogen, 71-0500), and GJPs were quantified as described in *Quantification of base-edited GJPs*.

### Evaluation of the physiological function of AAV-mediated base-edited GJPs.

To quantify ion permeability via gap junctions, monolayers of confluent cells were infected with the all-in-one AAV vector at a MOI of 5 × 10^5^ vg/cell and maintained in DMEM containing 10% FBS at 37°C and 5% CO_2_ for 72 hours. GJP function was quantified as described in *Scrape loading and dye transfer assay*.

### Ex vivo infection of the all-in-one AAV vector to cochlea tissue from the mouse model with GJB2 R75W.

Organotypic cochlear cultures were generated from the cochleae of P0 pups that had been sacrificed by decapitation and dissected in ice-cold PBS containing penicillin (100 IU/mL) (Gibco, 15140122). The epithelium of the organ of Corti was removed, and the explants were maintained in 8-well glass chambers (Iwaki, 5732-008) coated with 0.1% gelatin solution. Samples were submerged in 200 μL DMEM containing penicillin (100 IU/mL) and N2 supplement (×100) (Gibco, 17502048) and incubated at 37°C and 5% CO_2_ overnight to ensure proper adhesion to the glass of the chamber. Subsequently, samples were infected with the all-in-one AAV vector at 4.32 × 10^10^ vg/sample and maintained in DMEM containing penicillin (100 IU/mL), N2 supplement (×100), and 1% FBS. After incubation for 72 hours, the samples were fixed with 4% PFA in PBS and then permeabilized in PBS containing 0.1% Triton X-100. Samples were blocked with 2% BSA in PBS and incubated with anti-CX26 (1:300, Invitrogen, 71-0500) followed by incubation with Cy3-conjugated anti-rabbit IgG (1:1,000, Invitrogen, A10520), and then counterstained with DAPI (Vector Laboratories, H-1200). Fluorescence images were captured with a Zeiss Observer.Z1 microscope (Carl Zeiss), and fluorescence intensity was calculated with Keyence software.

### Auditory brain stem response.

All electrophysiological measurements were performed within a grounded test room that was acoustically and electrically insulated. For measurement of auditory brain stem response (ABR), mice were anesthetized and maintained in a head holder. Stainless-steel needle electrodes were placed at the vertex and ventrolateral to the left and right ears. The ABRs were measured using waveform storing and stimulus control with Scope software and the Power Lab system (PowerLab4/25, AD Instruments). Electrocardiograms were recorded using an extracellular AC Preamplifier (P-55, Astro-Med). Acoustic stimuli were delivered using a coupler type speaker (ES1spc, Bio Research Center). Thresholds were determined for click sounds and tone bursts (frequencies of 8 kHz) from a set of responses at different intensities (5 dB intervals). Electrical signals were averaged over 512 repetitions. Hearing thresholds of >95 dB were listed as 100 dB.

### In vivo AAV-mediated base editing for CX26^R75W+^ mice.

Injection of the all-in-one AAV vector was performed with some modification (15, 39). Nine- to 30-week-old CX26*^R75W+^* mice were anesthetized with an intraperitoneal injection of sodium pentobarbital (75 mg/kg). The AAV vector filled a Hamilton syringe and was injected into the perilymph through the lateral semicircular canal of the inner ear of left ear via a fused silica capillary tube (Eicom, 90000). A part of the lateral semicircular canal was opened with a dental drill, and 20 μL of the viral vector (4.32 × 10^12^ vg/mL) was injected into the perilymph at a rate of 3 μL/min using a microsyringe pump connected to the Hamilton syringe. To prevent the injected vector from leaking into the outside of the inner ear, the hole of semicircular canal was sealed by epithelial tissue from the treated mouse. Bleeding from the wound was prevented by BOLHEAL (KM Biologics, 07500701). The right ear was not treated. Two weeks after administration, mice were anesthetized and killed before the inner ear tissues were removed. Cochleae were dissected, fixed in 4% PFA, and then permeabilized in PBS containing 0.1% Triton X-100. Samples were blocked with 2% BSA in PBS and incubated with anti-CX26 (1:300, Invitrogen, 71-0500) followed by incubation with Cy3-conjugated anti-rabbit IgG (1:1,000, Invitrogen, A10520), and then counterstained with DAPI (Vector Laboratories, H-1200). Fluorescence images were captured with a Zeiss Observer.Z1 microscope (Carl Zeiss), and fluorescence intensity was calculated with Keyence software.

### Generation of GJB2 mutants derived from the bystander effect.

The formation of GJPs formed by *GJB2* mutants was evaluated by quantifying the transient expression of CX26 WT and its mutants (I74T, R75W, I74T&W77R, I74T&R75W, and I74T&L76P). Each mutation was introduced into the ORF of human *GJB2* cDNA in plasmid pcDNA3-GFP by PCR site-directed mutagenesis using the Agilent QuikChange kit (Agilent,200518). HeLa cells were transfected with plasmids using FuGENE HD transfection reagent (Promega, E2311) and cultured in DMEM containing 10% FBS. After 48 hours of incubation, the samples were fixed with 4% PFA in PBS and then permeabilized in PBS containing 0.1% Triton X-100. Samples were blocked with 2% BSA in PBS and incubated with Alexa Fluor 633–conjugated phalloidin (1:400, Invitrogen, A22284) and then counterstained with DAPI (Vector Laboratories, H-1200). Fluorescence images were captured with a Zeiss Observer.Z1 microscope (Carl Zeiss).

### Statistics.

The data are presented as the mean ± SEM. Differences were assessed using Mann-Whitney *U* test or Kruskal-Wallis test with Dunn’s multiple comparisons test using GraphPad Prism software 9. A *P* value of less than 0.05 was considered significant.

### Study approval.

All animal experimental procedures were conducted in accordance with the guidelines of and with the approval of the Juntendo University Animal Care and Use Committee (approval 2024-201). All mice were housed under specific pathogen–free conditions.

### Data availability.

Detailed information for each base-editing vector is available from VectorBuilder. The IDs for each vector are VB220620-1040dnx, VB221024-1475wfy, VB221024-1477xed, VB221024-1478jxf, VB221024-1479afb, and VB220620-1054pua. Values for all data points in graphs are reported in the Supporting Data Values file.

## Author contributions

TU, DA, RN, FM, KI, ON, and KK conceptualized the study. TU, DA, RN, FM, KI, ON, and KK devised the methodology. TU, DA, HT, and RN performed experiments and/or interpreted data. DA, RN, ON, and KK provided critical technical or material support. KK and ON designed and supervised the project. TU drafted the manuscript. KK acquired funding.

## Supplementary Material

Supplemental data

Unedited blot and gel images

Supporting data values

## Version 1 03/10/2025

Electronic publication
