# Supplementary material for: AAV-mediated base editing restores cochlear gap junction in GJB2 dominant-negative mutation-associated syndromic hearing loss model
Source: JCI Insight. 2025 Mar 10;10(5):e185193. doi: 10.1172/jci.insight.185193 (PMC11949026; doi:10.1172/jci.insight.185193)
Supplement: Supplemental data [file jciinsight-10-185193-s297.pdf]

Supplemental Figure 1

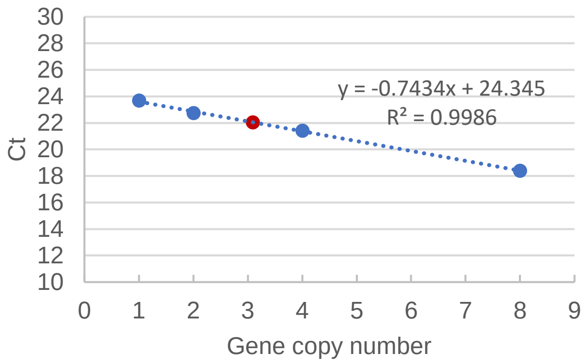

Standerd curve		HeLa CX26 R75W	
Ct	Copy number	Ct	Copy number
23.6830	1	22.0465	[3.09] = 3
22.7369	2		
22.1155	3		
21.4119	4		
18.3980	8		

Supplemental Figure 1. Determination of transgene copy number. Copy number of the *GJB2* R75W transgene in the genome of HeLa cell was validated by a real-time qPCR (n=3). Standard curves were prepared from genomic DNA of non-transfected HeLa cells and synthetic gene encoding CMV promoter and *GJB2* R75W. Four serial dilutions were prepared in the range from 100-100,000 cells for genomic DNA and from 100-100,000 copies for synthetic gene. Genomic DNA from HeLa cells expressing the CX26 R75W mutation (sample) was also prepared in four serial dilutions in the same range as for the standard curve of genomic DNA of non-transfected HeLa cells. The primers were designed in the CMV promoter region upstream of *GJB2* gene. CMV F (universal): CGCAAATGGGCGGTAGGCGTG, R: GTAAGCAGTGGGTTCTCTAG. Real-time qPCR reactions were performed with the TB Green Premix Ex Taq II (Takara) with a real-time PCR system (Applied Biosystems ABI Step one plus) and analyzed with Step one software (Applied Biosystems: Step one software version 2.3).

Supplemental Figure 2

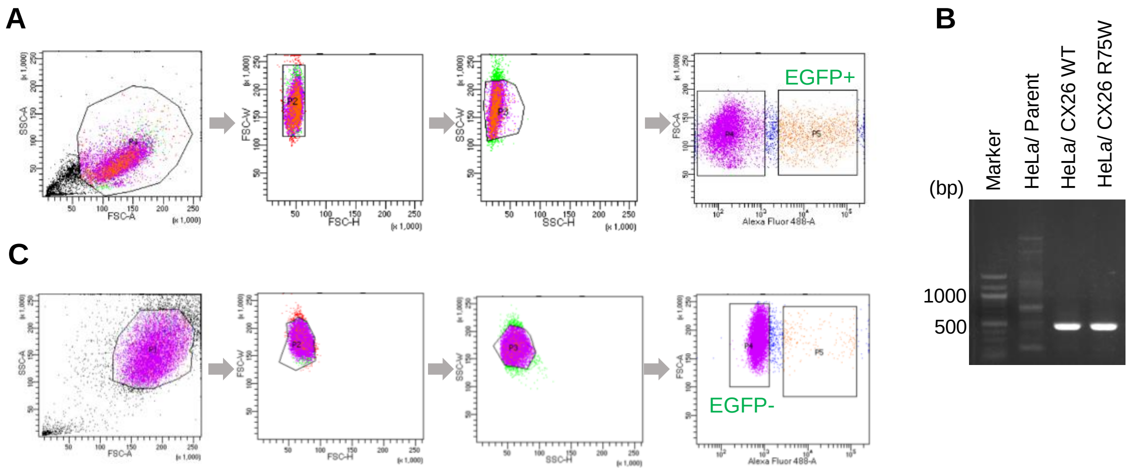

Supplemental Figure 2. Gating strategies for flow cytometry. (A) EGFP-positive cells were sorted. **(B)** Genomic DNA was extracted, and *GJB2* transgene was amplified by PCR. **(C)** EGFP-negative cells were sorted.

Supplemental Figure 3

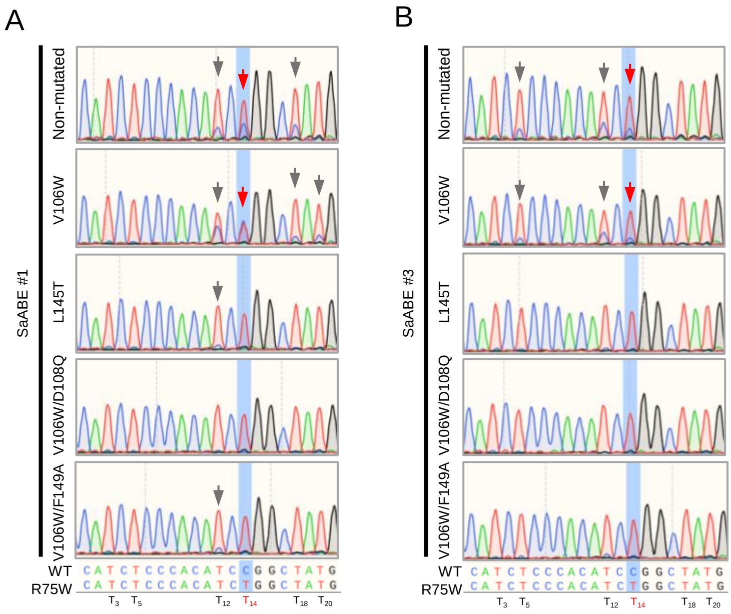

Supplemental Figure 3. Representative results from direct genome sequencing of the gene correction from SaABE with the TadA8e mutants. Base-editing efficacy with SaABE#1 (A) or SaABE#3 (B).

Supplemental Figure 4

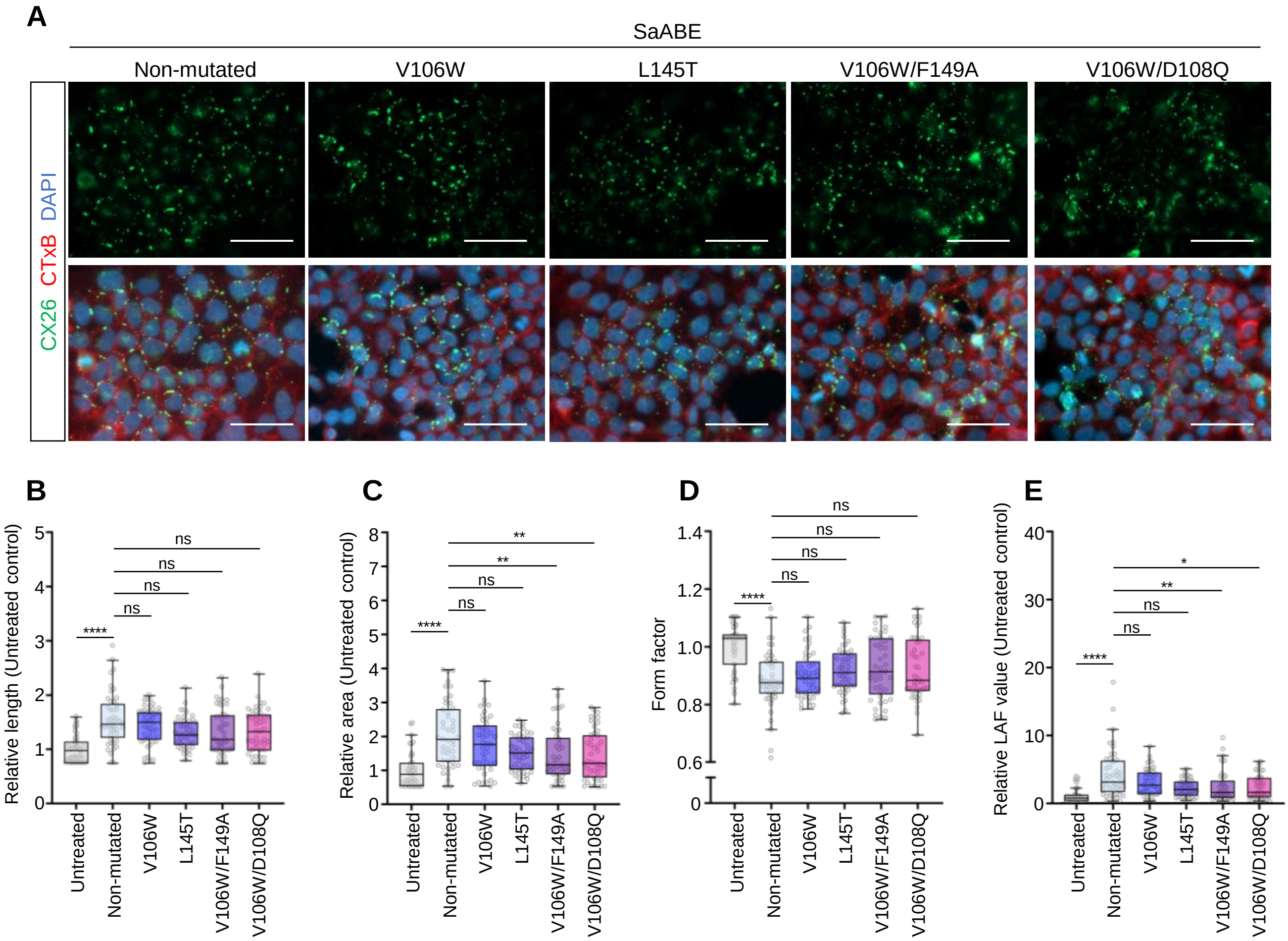

Supplemental Figure 4. Optimization of base-editing vectors. (**A**) Representative images of GJPs (GJPs) after base editing by SaABE#3 including the TadA8e mutants. Scale bar: 20 μm. (**B–E**) Quantitative data of length (**B**), area (**C**), form factor (**D**) and LAF (**E**) presented in panel **A**. Box-and-whisker plot shows median, interquartile range, minimum and maximum values, isolated dots beyond the whiskers correspond to outliers defined as a value that is smaller than the lower quartile −1.5 × the interquartile range or larger than the upper quartile +1.5 times the interquartile range. Each value was normalized to the value obtained for HeLa/CX26 R75W cells (untreated). n= at least 43 per group. Statistical significance was determined with Kruskal–Wallis test with Dunn's multiple comparisons test. * $P < 0.05$, ** $P < 0.01$ and **** $P < 0.0001$.

Supplemental Figure 5

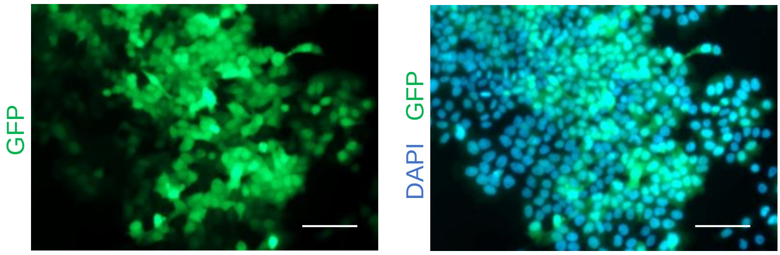

Supplemental Figure 5. Tropism of AAV-Sia6e for HeLa cells. HeLa cells were infected with AAV-Sia6e vector containing EGFP at a MOI of 5×10^5 viral genomes per cell and maintained in DMEM containing 10% FBS at 37°C and 5% CO_2. After incubation for 48 h, cells were counterstained with DAPI. Fluorescence images were acquired with a Zeiss Observer.Z1 microscope. EGFP: green; nucleus: cyan. Scale bar: 100 µm.
