# Supplementary figures and images for: AAV-mediated base editing restores cochlear gap junction in GJB2 dominant-negative mutation-associated syndromic hearing loss model
Source: JCI Insight. 2025 Mar 10;10(5):e185193. doi: 10.1172/jci.insight.185193 (PMC11949026; doi:10.1172/jci.insight.185193)

Full unedited blotgel for Supplementary Figure 2B

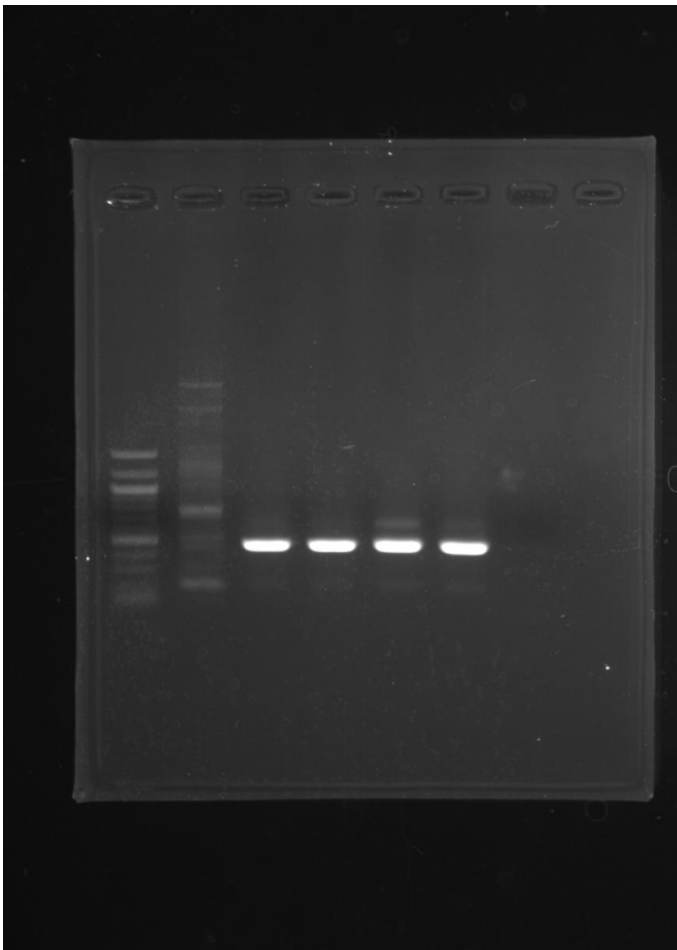

Supplement: Unedited blot and gel images [file jciinsight-10-185193-s298.pdf]
